# Supplementary material for: Modeling glioblastoma heterogeneity as a dynamic network of cell states
Source: Mol Syst Biol. 2021 Sep 16;17(9):e10105. doi: 10.15252/msb.202010105 (PMC8444284; doi:10.15252/msb.202010105)
Supplement: Supplementary file 6 — Source Data for Figure 5 [file MSB-17-e10105-s004.zip › Figure5A_sourcedata/GSEA_3071/nosigdb_stateV.GseaPreranked.1622795159954/LARSSON_STATE_1.html]

Details for gene set LARSSON\_STATE\_1[GSEA]

|  || Dataset | state43071 |
| Phenotype | NoPhenotypeAvailable |
| Upregulated in class | na\_neg |
| GeneSet | LARSSON\_STATE\_1 |
| Enrichment Score (ES) | -0.49257922 |
| Normalized Enrichment Score (NES) | -2.564191 |
| Nominal p-value | 0.0 |
| FDR q-value | 0.0 |
| FWER p-Value | 0.0 |
Table: GSEA Results Summary

  

Fig 1: Enrichment plot: LARSSON\_STATE\_1      
 Profile of the Running ES Score & Positions of GeneSet Members on the Rank Ordered List

  

| PROBE | GENE SYMBOL | GENE\_TITLE | RANK IN GENE LIST | RANK METRIC SCORE | RUNNING ES | CORE ENRICHMENT || 1 | GAP43 |  |  | 61 | 1.501 | 0.0143 | No |
| 2 | IGFBP3 |  |  | 78 | 1.437 | 0.0362 | No |
| 3 | CSRP2 |  |  | 90 | 1.396 | 0.0583 | No |
| 4 | TMEFF2 |  |  | 224 | 1.142 | 0.0526 | No |
| 5 | STK17A |  |  | 493 | 0.874 | 0.0165 | No |
| 6 | CTNNBIP1 |  |  | 509 | 0.857 | 0.0285 | No |
| 7 | UBE2S |  |  | 514 | 0.854 | 0.0425 | No |
| 8 | GCHFR |  |  | 519 | 0.852 | 0.0565 | No |
| 9 | SCG2 |  |  | 682 | 0.756 | 0.0386 | No |
| 10 | DPYSL3 |  |  | 691 | 0.750 | 0.0501 | No |
| 11 | NETO2 |  |  | 717 | 0.740 | 0.0581 | No |
| 12 | PMEPA1 |  |  | 718 | 0.740 | 0.0709 | No |
| 13 | PTTG1 |  |  | 936 | 0.647 | 0.0406 | No |
| 14 | MAP3K7CL |  |  | 1055 | 0.606 | 0.0285 | No |
| 15 | CCDC85B |  |  | 1169 | 0.572 | 0.0168 | No |
| 16 | AP1S2 |  |  | 1205 | 0.560 | 0.0198 | No |
| 17 | PDLIM4 |  |  | 1382 | 0.511 | -0.0050 | No |
| 18 | TUBB4B |  |  | 1659 | 0.444 | -0.0501 | No |
| 19 | S100A16 |  |  | 1824 | 0.414 | -0.0743 | No |
| 20 | ANXA1 |  |  | 2165 | 0.366 | -0.1330 | No |
| 21 | ARPC1A |  |  | 2335 | 0.344 | -0.1594 | No |
| 22 | TMEM158 |  |  | 2340 | 0.343 | -0.1542 | No |
| 23 | CTSV |  |  | 2541 | 0.317 | -0.1870 | No |
| 24 | OAZ1 |  |  | 3291 | -0.264 | -0.3257 | No |
| 25 | WDR1 |  |  | 3447 | -0.289 | -0.3503 | No |
| 26 | TPM4 |  |  | 3591 | -0.314 | -0.3722 | No |
| 27 | PRELID1 |  |  | 3709 | -0.332 | -0.3889 | No |
| 28 | RPL8 |  |  | 3733 | -0.336 | -0.3874 | No |
| 29 | SYNJ2 |  |  | 3787 | -0.347 | -0.3916 | No |
| 30 | TUBA1C |  |  | 3957 | -0.379 | -0.4173 | No |
| 31 | TXNRD1 |  |  | 4077 | -0.404 | -0.4331 | No |
| 32 | LDHA |  |  | 4145 | -0.421 | -0.4386 | No |
| 33 | SPATS2L |  |  | 4148 | -0.422 | -0.4317 | No |
| 34 | USP53 |  |  | 4297 | -0.472 | -0.4519 | No |
| 35 | PMAIP1 |  |  | 4312 | -0.476 | -0.4463 | No |
| 36 | PLP2 |  |  | 4366 | -0.490 | -0.4479 | No |
| 37 | DSTN |  |  | 4560 | -0.570 | -0.4750 | No |
| 38 | SRM |  |  | 4653 | -0.620 | -0.4818 | Yes |
| 39 | CDC42EP3 |  |  | 4669 | -0.627 | -0.4739 | Yes |
| 40 | TMSB10 |  |  | 4688 | -0.639 | -0.4662 | Yes |
| 41 | LGALS1 |  |  | 4784 | -0.699 | -0.4723 | Yes |
| 42 | S100A13 |  |  | 4791 | -0.705 | -0.4612 | Yes |
| 43 | EIF6 |  |  | 4817 | -0.730 | -0.4534 | Yes |
| 44 | PKM |  |  | 4828 | -0.737 | -0.4425 | Yes |
| 45 | SMS |  |  | 4842 | -0.750 | -0.4320 | Yes |
| 46 | RPS12 |  |  | 4888 | -0.795 | -0.4268 | Yes |
| 47 | CLIC1 |  |  | 4899 | -0.809 | -0.4147 | Yes |
| 48 | PLIN3 |  |  | 4921 | -0.833 | -0.4043 | Yes |
| 49 | C12orf75 |  |  | 4940 | -0.858 | -0.3929 | Yes |
| 50 | HMGA1 |  |  | 4962 | -0.878 | -0.3817 | Yes |
| 51 | IGFBP5 |  |  | 4978 | -0.897 | -0.3690 | Yes |
| 52 | S100A10 |  |  | 5011 | -0.954 | -0.3586 | Yes |
| 53 | ACTG1 |  |  | 5017 | -0.961 | -0.3429 | Yes |
| 54 | TXN |  |  | 5054 | -1.035 | -0.3319 | Yes |
| 55 | ANXA2 |  |  | 5055 | -1.036 | -0.3139 | Yes |
| 56 | TWIST1 |  |  | 5060 | -1.052 | -0.2965 | Yes |
| 57 | SH3BGRL3 |  |  | 5063 | -1.064 | -0.2784 | Yes |
| 58 | CAPN2 |  |  | 5088 | -1.120 | -0.2636 | Yes |
| 59 | CAVIN1 |  |  | 5116 | -1.195 | -0.2480 | Yes |
| 60 | COTL1 |  |  | 5120 | -1.206 | -0.2277 | Yes |
| 61 | LMNA |  |  | 5141 | -1.277 | -0.2094 | Yes |
| 62 | CAST |  |  | 5142 | -1.277 | -0.1873 | Yes |
| 63 | ARL4C |  |  | 5143 | -1.279 | -0.1651 | Yes |
| 64 | S100A6 |  |  | 5183 | -1.473 | -0.1471 | Yes |
| 65 | PYGB |  |  | 5187 | -1.486 | -0.1219 | Yes |
| 66 | S100A11 |  |  | 5196 | -1.536 | -0.0968 | Yes |
| 67 | NQO1 |  |  | 5210 | -1.596 | -0.0716 | Yes |
| 68 | SPP1 |  |  | 5272 | -2.446 | -0.0409 | Yes |
| 69 | CAV1 |  |  | 5279 | -2.605 | 0.0031 | Yes |
Table: GSEA details [plain text format]

  

Fig 2: LARSSON\_STATE\_1: Random ES distribution      
 Gene set null distribution of ES for **LARSSON\_STATE\_1**
